# “The Social Network” and Muscular Dystrophies: The Lesson Learnt about the Niche Environment as a Target for Therapeutic Strategies

**DOI:** 10.3390/cells9071659

**Published:** 2020-07-09

**Authors:** Ornella Cappellari, Paola Mantuano, Annamaria De Luca

**Affiliations:** Section of Pharmacology, Department of Pharmacy-Drug Sciences, University of Bari “Aldo Moro”, via Orabona 4—Campus, 70125 Bari, Italy; ornella.cappellari@uniba.it (O.C.); paola.mantuano@uniba.it (P.M.)

**Keywords:** muscle regeneration, muscle stem cells, stem cells niche, muscle homeostasis, neuromuscular disorders, Duchenne muscular dystrophy, pharmacological approach

## Abstract

The muscle stem cells niche is essential in neuromuscular disorders. Muscle injury and myofiber death are the main triggers of muscle regeneration via satellite cell activation. However, in degenerative diseases such as muscular dystrophy, regeneration still keep elusive. In these pathologies, stem cell loss occurs over time, and missing signals limiting damaged tissue from activating the regenerative process can be envisaged. It is unclear what comes first: the lack of regeneration due to satellite cell defects, their pool exhaustion for degeneration/regeneration cycles, or the inhibitory mechanisms caused by muscle damage and fibrosis mediators. Herein, Duchenne muscular dystrophy has been taken as a paradigm, as several drugs have been tested at the preclinical and clinical levels, targeting secondary events in the complex pathogenesis derived from lack of dystrophin. We focused on the crucial roles that pro-inflammatory and pro-fibrotic cytokines play in triggering muscle necrosis after damage and stimulating satellite cell activation and self-renewal, along with growth and mechanical factors. These processes contribute to regeneration and niche maintenance. We review the main effects of drugs on regeneration biomarkers to assess whether targeting pathogenic events can help to protect niche homeostasis and enhance regeneration efficiency other than protecting newly formed fibers from further damage.

## 1. The Muscle Tissue: Development and Insight

Skeletal muscle is a complex and heterogeneous tissue with a high regeneration potential and plasticity. Muscle regeneration recapitulates skeletal muscle ontogenesis for many aspects. Myogenesis can be divided into different phases, which comprehend embryonic (from E10.5 to E12.5 of mouse development) and fetal (from E14.5 to E17.5) phases [1]. First, muscle fibers are generated during embryonic myogenesis in the somites, transient mesodermal units, to which other fibers are subsequentially added for following differentiation into ventral sclerotome and a dorsal dermomyotome [2]. Myogenic progenitors appear at the end of the somitogenesis and respond to signals from the neural tube, such as Wnts (wingless-type MMTV integration site family) and Sonic hedgehog (Shh), which activate the basic helix–loop–helix transcription factors, such as myogenic factor 5 (Myf5) and myoblast determination protein 1 (MyoD) which commit cells to myogenesis [3]. The embryology of skeletal muscle is out of the scope of this review and excellent reviews on the topic are available [3].

Importantly, as previously stated, skeletal muscle is formed in successive and distinct, though overlapping waves, involving different types of myoblasts (embryonic, fetal myoblasts, and satellite cells). The progressive growth of muscles occurring during late embryonic (E10.5–12.5), fetal (E14.5–17.5), and postnatal life was recently attributed to a population of muscle progenitors that can be found already at embryonic stage [4,5,6,7]. These might derive from a paired box gene (*Pax*) *3/7* positive population of myogenic progenitors, residing in the central part of the dermomyotome. Around E11.5 of mouse development, embryonic myoblasts enter the myotome and fuse into myotubes. More or less at the same stage, during a phase referred to as primary myogenesis, myogenic progenitors (migrated from the dermomyotome to the limb), start to differentiate into multinucleated muscle fibers, commonly known as primary fibers. A second wave of myogenesis (from E14.5 and E17.5 in mouse) known as secondary myogenesis, is characterized by fetal myoblasts fusing with each other [8,9,10]. At the end of this phase, satellite cells can be morphologically identified as mononucleated cells located between the basal lamina and the sarcolemma. During perinatal and also postnatal development, satellite cells start dividing at a slow pace. Most of the progeny fuse with the adjacent fibers, with new nuclei contributing to growing muscle fibers (whose nuclei are not able to divide). Because of this process, it is possible to think that the majority of the nuclei of a mature muscle are probably derived from satellite cells. Then, when postnatal growth is finished, satellite cells enter a phase of quiescence, but they can be activated when the muscle tissue is damaged or in response to further growth demands. In these cases, satellite cells exit the quiescent state, and undergo a number of cells divisions, thereby producing fusion competent cells that are able either to fuse with damaged fibers or to form new ones. Moreover, part of the cells return instead to quiescence, thereby maintaining the progenitor pool. This ability has led to the suggestion that they represent a type of stem cells [11]. Many factors influence satellite cells’ population during myogenesis, such as obesity, diabetes, and other metabolism-related problems. A very important one, for example, is represented by nutrient administration in the maternal stage, which seems to have a direct role in perinatal muscle growth, as extensively explained in Fiorotto and Davis [12].

## 2. Muscle Stem Cell Niche: Role in Tissue Homeostasis and Muscle Regeneration

Satellite cells occupy an exclusive niche within the muscle tissue, with both stem-like properties and demonstrated myogenic activities. As previously stated, satellite cells are able to remain quiescent or they can be activated in response either to growth/regenerative signal/injuries [13]. After this activation, they re-enter the cell cycle and undergo an asymmetric division to maintain self-renewal. Self-renewal is perpetuated via symmetric cell expansion (generating two identical daughter stem cells) or through an asymmetric cell division (generating both a stem cell and a committed progenitor daughter cell) [14].

Of the two formed daughter cells, one goes back replenishing the niche, then becoming quiescent again; meanwhile, the other participates in the muscle regeneration/growth/homeostasis process. This mechanism is finely regulated. In fact, satellite cell fate is tuned by mechanisms involving both cell-autonomous and external stimuli, in concert with the programmed expression and action of various transcription factors [15,16]. The complex processes governing satellite cell activation and myogenesis have attracted much interest over the years and have been beautifully revised [16,17]. Notably, the decision to undergo symmetric or asymmetric self-renewal is a critical step in satellite cell fate determination, and a deregulation of this process could potentially have detrimental consequences on the execution of a muscle regeneration program. Satellite cells are located beneath the basal lamina in a quiescent state, in which they express Pax7 and Myf5 [18]. When they are activated and differentiate into myoblasts, they express MyoD and myogenin (Myog). If a Pax7+ cell population is deleted, skeletal muscle regeneration is impaired, thereby reinforcing the importance of these cells in this process [19]. After muscle injury, there is a time-dependent and well-organized inflammatory response that happens together with satellite cell activation and through their differentiation process. The recruitment of immune cells to the site of injury is pivotal to obtaining complete skeletal muscle regeneration [20]. The acute inflammatory response following muscle injury usually begins with neutrophils infiltration [21]. This is usually followed by an infiltration of macrophages carrying an M1 phenotype, which produces mostly inflammatory cytokines, such as tumor necrosis factor-alpha (TNF-α), interleukin 1 beta (IL-1β), and interferon-gamma (IFN-γ) [22].

The addition of these cytokines in primary myoblasts’ culture remarkably increases cell proliferation, supporting the assumption that the early increase in M1 macrophage population and the first phase of inflammation actively participate in satellite cell activation [23]. Afterward, an important expansion of M2 macrophages does occur, which is associated with tissue repair and satellite cell differentiation [24]. In fact, M2 macrophages produce different cytokines, such as interleukins IL-4 and IL-10, which improve myoblast differentiation in vitro and increase Myog expression levels, the transcription factor that is essential for satellite cell terminal differentiation [25]. Therefore, M1 and M2 macrophages’ kinetics are critical for the early steps of muscle regeneration.

Epigenetic regulation mechanisms can also play a role in satellite cell pool maintenance by modulating proliferation and differentiation. A complete epigenetic profiling of quiescent satellite cells, obtained by Liu and colleagues via chip-seq analysis, has shown that during quiescence, chromatin is maintained in a transcriptionally permissive state, thereby allowing various epigenetic modifications, leading to increased expression of genes involved in satellite cells’ proliferation. In particular, DNA methylation of some genes promoters (e.g., *Notch*, Notch homolog 1, translocation-associated), has been found to cause changes in satellite cell renewal, maintenance, and homeostasis [26,27,28].

In addition, mitochondrial functions, particularly fission and fusion, have been recently reported to play a role in maintaining and dictating satellite cells’ fates. In fact, mitochondria are strongly connected to metabolic programming during quiescence, activation, self-renewal, proliferation, and differentiation. Interestingly, mitochondrial adaptation might take place to modify satellite cells’ fates and function in the presence of different environmental cues, and under different metabolic states [29,30]. Therefore, satellite cells’ functional outcomes are strongly associated with mitochondrial energy output [31,32]. Mitochondrial functions are so broad that some of them, including regeneration, could be interesting targets for pharmacological therapy.

However, while muscle repair after damage is an efficient process in healthy muscle, its probability of success appears low in many muscle disorders. The maintenance of an efficient regeneration process is guaranteed by both satellite cells’ niche environment and satellite cells’ pool. By disrupting either one of the two or both, the impairment in muscle regeneration suddenly happens, as likely occurs in many muscular dystrophies. In Duchenne muscular dystrophy (DMD), the most frequent and most studied one, it is still unclear which phenomenon comes first, also in relation to the different roles that cytokines play on adult myofiber and satellite cells and their complex crosstalk. However, the plethora of data collected in DMD over the last decades, also with the extensive use and characterization of the *mdx* mouse, its main animal model [33]. allow a deeper insight into the possibility to improve regeneration efficiency as a consequence of therapeutic approaches, from classical drugs to cell therapy.

Pharmacological approaches, even if unable to restore the primary defect, can target disease pathophysiology and progression, by acting at different stages of the inflammatory cascade and therefore slowing down the necrotic process. Yet, the outcomes of such strategies on regeneration efficiency are still unclear. Similarly, other approaches aim at enhancing regeneration with a direct drug action on satellite cell activation and differentiation, although such an effect without a parallel mechanism to minimize the damage of mature myofiber appears to be weak. Herein, we critically revised some of the main approaches used preclinically and clinically in DMD in the attempt to assess their potential outcomes in maintaining or enhancing the regeneration potential, also in relation to the mechanism of action. The overview of these effects may help the community to go back to basic scientific research with a better understanding of the imbalance in the social network governing muscle repair and stem cell niche in relation to disease mechanisms to better address therapeutic intervention for tissue repair. 

## 3. Duchenne Muscular Dystrophy: Is There a Role for Satellite Cells and Their Niche?

### 3.1. DMD General Picture

DMD is a lethal progressive pediatric muscle disorder. It is genetically inherited as an X-linked disease caused by mutations in the dystrophin gene. The *DMD* gene (which encodes the protein dystrophin) is affected by point mutations, duplications, and deletions of parts of the gene, causing alterations in the reading frame and consequent truncation of the dystrophin protein, which is then rapidly degraded. Dystrophin protein is mainly expressed in skeletal and cardiac muscle and to a lesser extent in smooth muscle and the brain. Dystrophin represents an essential component of the large oligomeric dystrophin-glycoprotein complex (DGC) [34,35]. The DGC acts as a connector between the actin cytoskeleton of the myofiber and the surrounding extracellular matrix (ECM) through the sarcolemma. In the absence of dystrophin, DGC assembly is impaired which weakens the muscle fibers, rendering them highly susceptible to injury. At this point, muscle contraction-induced stress results in constant cycles of degeneration and regeneration [36]. Eventual accumulation of inflammation and fibrosis lead to progressive muscle weakening and loss of muscle mass and function [37]. The efficiency of regeneration appears to be low [38]. This progressive muscle wasting condition leads to severe disability and follows with premature death in affected individuals due to respiratory and/or cardiac failure, typically by or before the age of 30.

### 3.2. DMD and Stem Cell Polarity

DMD has also been considered a stem cell disease, as a failing regeneration is a typical feature. In fact, there is still a debate about the main determinant of the regeneration failure; it is not clear whether the lack of dystrophin impairs satellite cells’ ability to repair the muscle, or the disruption of the stem cells niche environment, or the two altogether. The role of the niche for stem cells in muscle repair is, in general, crucial. In the case of DMD, the niche environment is believed to be compromised by the cascade of events due to constant inflammation and muscle degeneration [39]. However, the absence of dystrophin can play a key role, by affecting asymmetric division. In general, cell cortex polarization and specification of the mitotic spindle orientation are critical steps for the asymmetric localization of cell fate determinants [40].

Importantly, dystrophin interacts with the cell polarity-regulating serine/threonine-protein kinase MARK2 at the muscle fiber membrane [41]. Moreover, it has been demonstrated that activated satellite cells also express dystrophin [42]. Interestingly, dystrophin protein expression has been found to be polarized in satellite cells, and apparently, it is expressed at a very high level when cells are about to undergo cell division. Therefore, dystrophin has a pivotal role in regulating polarity in asymmetric satellite cell division. In support of this view, Chang et al. described a significant reduction in asymmetric division, with a consequent progressive loss of myogenic progenitor, in myoblast-derived satellite cells isolated from *mdx* mouse [43]. This phenomenon seems due to the *mdx*-derived loss of polarity of *mdx* satellite cells, which then resulted in defective mitotic spindle orientation, causing an impairment in asymmetric cell division. This observation supports the hypothesis that the absence of dystrophin, even at the satellite cell level and during asymmetric division, is a significant contributing factor in the failing repair efficiency manifestation of DMD phenotype [43].

However, the niche surrounding environment can also play a key role in the effectiveness of regeneration. This will be addressed in the following paragraph. An important question that remains unaddressed is to determine what the fate is of dystrophic satellite cells that are unable to undergo proper cell division. In accordance with the observation that *mdx* satellite cells exhibit a reduced ability to commit to the myogenic program, another recent study found that satellite cells from *mdx* mice have reduced myogenic potential and initiate a fibrogenic program. It is conceivable that satellite cell dysfunction in DMD can also account for enhanced fibrosis.

### 3.3. Inflammation and Regeneration Efficiency in DMD

In DMD, muscle tissue goes through continuous cycles of degeneration/regeneration. In the latest stages of the pathology, muscle tissue is substituted by fibrotic and adipose tissue mostly due to the inability of satellite cells to repair muscle damage. Chronic inflammation is a typical hallmark of DMD and may contribute to impaired skeletal muscle regeneration. Although many cells are involved in chronic inflammation, one of the most important roles is played by macrophages, since these cells are associated not only to satellite cells’ activation but also to the survival of fibro/adipogenic progenitors (FAPs), which outcompete the satellite cell population during inflammation [44]. By competing with satellite cells, FAPs can increment the fibrotic process; an imbalance between the two populations ultimately leads to the accumulation of FAPs in skeletal muscles with consequent aberrant production of pro-fibrotic factors (e.g., ECM components). Thus, a pharmacological reduction of FAP accumulation could potentially help in preserving satellite cells and their ability to repair injured muscles.

Adding to the role of macrophages in modulating satellite cell proliferation and differentiation, a recent study demonstrated that the cytokines, produced by both M1 and M2, infiltrated macrophages in the injured skeletal muscle, are able to modulate ECM production through FAPs [45]. In particular, it has been shown that in physiological condition, ECM components’ production by FAPs was regulated by TNF-α or transforming growth factor-beta 1 (TGF-β1), which are both secreted by M1 and M2 macrophages. On top of that, the M1 and M2 macrophage kinetics after muscle damage supported the initial accumulation followed by FAP apoptosis, avoiding the aberrant deposition of ECM in skeletal muscle. In this regard, an increase in both cytokines can be responsible for excessive ECM accumulation, thereby leading to poor or non-effective skeletal muscle regeneration. Based on those previous studies, FAPs and macrophages have been characterized as some of cells associated with generation and maintenance of the microenvironment responsible for satellite cells’ activation and differentiation, i.e., the satellite cell niche, pivotal during skeletal muscle regeneration process. Even though the acute inflammatory response is associated with proper skeletal muscle regeneration, chronic and non-resolute inflammation, which is observed in the skeletal muscle of idiopathic inflammatory myopathies, dystrophies, and aging, is strongly associated with the impaired functions of satellite cells, immune cells, and FAPs, leading then to fibrosis accumulation and poor skeletal muscle regeneration [45,46].

Regarding M1 macrophage over-activation, some in vitro studies show that higher levels of the cytokines produced by these cells (e.g., IL-1β, TNF-α, IFN-γ) are able to mitigate or abrogate myoblast proliferation [23,47]. Moreover, the continuous stimulation of myogenic progenitor cells by IFN-γ leads to the suppression of genes responsible for terminal differentiation. This suppression accounts for the activity of the histone methyltransferase EZH2, which is mediated by the class II major histocompatibility complex transactivator [48]. A chronic increase in IFN-γ and class II major histocompatibility complex transactivator levels repress the expression of genes related to the late stages of satellite cell differentiation by enhancing the promoter region of these genes [48]. Although the level and chronicity of IFN-γ required to start these epigenetics effects in vivo are unknown, these findings suggest that a persistent increase in M1 macrophages expressing IFN-γ for a long time can definitely impair skeletal muscle regeneration. Table 1 summarizes the main cytokines involved in the early and late damaging signals, and in the pro-fibrotic pathways.

## 4. Pharmacological Approaches Targeting Niche Homeostasis: What We Learned from DMD

For many years, scientists have been putting a lot of effort into finding an effective and definitive treatment for DMD patients. Although pharmacological and technological progress has been made, there is still no absolute cure for this severe disease. Several promising gene and molecular therapies are currently under investigation, aimed at targeting the primary defect. These include gene replacement, exon skipping, and suppression of stop codons [56,57,58]. More recently, the promising gene-editing tool CRISPR/Cas9 has been offering exciting perspectives for restoring dystrophin expression in patients with DMD [59,60,61].

In parallel, various therapeutic strategies have been explored with drugs able to target the complex secondary mechanisms responsible for DMD pathogenesis. The aim is to find drugs safer than the current standard of care represented by corticosteroids. Indeed, glucocorticoids are beneficial to prolonging ambulation in DMD boys and are initiated early before other symptomatic therapies. The main efficacy of steroids is believed to be related to the control of inflammation [62,63,64,65].

Along this line, a large number of drugs have been investigated in DMD, many of them aimed at reducing inflammation and fibrosis, and they are then able to shut down the pathological loop leading to progressive damage [58]. Among this surge of new experimental pharmacotherapies, in this review, we will revise available data to assess whether drugs can help to maintain a proper equilibrium in satellite cell self-renewal via direct action, or mainly by regulating the inflammatory response and controlling fibrosis. In fact, to date, it is still unclear whether there is a pharmacological treatment that can help in maintaining better muscle homeostasis and improving satellite cell efficiency.

### 4.1. Biomarkers of Regeneration in DMD: Advantages and Limitations

From the perspective of evaluating the potential ability of old and new pharmacological strategies to modulate skeletal muscle regeneration in DMD, it is crucial to rely on a robust panel of translational biomarkers to obtain a more complete view on and assessment of the regenerative process in preclinical research. Recently, besides several valuable indices commonly used to quantify regeneration, degeneration, and repair efficiency, the advances in technology offered many possibilities to implement the number of regenerative biomarkers to be assessed. All the identified biomarkers described in this paragraph are summarized in Table 2. 

The histological evaluation of dystrophic muscles in DMD patients and animal models is the most traditional way to quantify and characterize damage and regeneration. The classical and standardized hematoxylin and eosin (H&E) staining protocol (TREAT-NMD Standard Operating Procedures for the *mdx* mouse model; DMD_M.1.2.007) enables evaluating histopathology. In this context, the proportion of centronucleated fibers (CNF) represents a common index of regeneration, in parallel to a morphological change in size of nascent muscle cells (DMD_M.1.2.001) [66]. However, this structural assessment of the regenerative process is characterized by intrinsic limitations and variables which can complicate data interpretation; e.g., concerning how the level of centronucleation is associated with a still efficient/non-efficient repair system, the identification of activated satellite cells and the amount of fibrosis depending on pathology stage. Part of these uncertainties can be solved with immunohistochemistry (IHC) and immunofluorescence (IF) techniques that allow us to appreciate the indices of satellite cell activation and regeneration, and the presence of specific markers of regeneration in fused myotubes. In this frame of knowledge, a robust regenerative biomarker in different muscles of *mdx* mice at different ages is the presence of developmental myosin heavy chains (embryonic and neonatal MyHCs), usually assessed by IF imaging [67].

High levels of MyHCs are also considered valuable indicators of disease severity which correlate well with functional impairment in DMD boys. However, this index is also subjected to misinterpretation, since MyHCs may be occasionally present in non-regenerating fibers and are differentially expressed throughout the regeneration process. IHC and IF can allow detecting the presence and cellular localization of any cytokine and transcription factor potentially involved in regeneration, and then help to quickly characterize the efficiency and extent of the process in natural disease history and as effects of therapeutics. qRT-PCR and gene array platforms, together with immunoblotting, ELISA, and proteomic arrays, are also widely used to detect regeneration biomarkers while researchers are intending to gain better insight into the mechanisms behind the regenerative process in DMD, and to assess whether drugs can modulate the expression of these indicators of regeneration in DMD. The transcription factor Pax7 is frequently assessed with various imaging and quantitative approaches, since its expression is directly related to the maintenance of the satellite cell pool, in parallel with the relative expression of other myogenic regulatory factors Myf5, MyoD, and Myog, in relation to the stage of the regenerative process [68,69].

A regeneration-associated biomarker of growing interest is represented by utrophin, an autosomal analogue of dystrophin (80% similarity between the two proteins), physiologically abundant in early developing muscles, and progressively replaced by dystrophin at the sarcolemma level towards birth. In DMD and *mdx* muscles, utrophin is upregulated because of the repairing process, but not to the extent to efficiently compensate for dystrophin absence [68]. As pointed out recently, utrophin sarcolemmal localization and the homogeneity of its signal across the whole muscle in correlation with ongoing regeneration, are critical to assess potential protection resulting from direct or indirect stimulation of its upregulation. Thus, the importance of combining imaging techniques to identify utrophin located at myofibers sarcolemma with the traditional assessment of regenerating fibers and their size is increasing [67,68,70,71].

Furthermore, since inflammation is crucial in modulating the muscle regeneration microenvironment, a more detailed view of the ongoing inflammatory process can be obtained by checking cytokine expression and their intracellular signaling, and by the parallel assessment and relative proportion of M1 and M2 macrophage phenotypes in relation to a drug treatment [68,72]. In parallel, considering the existing cross-talk between myogenesis and angiogenesis during muscle regeneration, orchestrated by restorative macrophages in vivo, the levels of growth factors and particularly of vascular endothelial growth factor (VEGF) along with its receptors, represent another set of biomarkers of interest to monitoring the progression of the microvasculature [73].

The multifunctional cell-surface protein neural cell adhesion molecule (NCAM) is expressed in activated satellite cells and during myogenic differentiation, representing a useful tool to evaluate active muscle regeneration following spontaneous and/or induced degeneration, and the proportion of adult myogenic cells already committed to differentiation [74,75].

Blood-circulating biomarkers are also becoming increasingly attractive for monitoring DMD disease progression and the efficacy of experimental therapeutic options. Among these, an emerging candidate for evaluating muscle regeneration in dystrophic animal models and DMD patients is serum osteopontin (OPN), an inflammatory cytokine and myogenic factor which has been recently found to be highly elevated in the early disease phase of CXMD_J_ (canine X-linked muscular dystrophy) dogs in Japan. Importantly, high serum OPN levels correlate well with phenotypic severity in CXMD_J_ dogs [76,77]. Similarly, in DMD patients, a single-nucleotide polymorphism (SNP, rs28357094T>G referred to as the G allele) in the promoter of OPN gene *SPP1* has been identified as a genetic modifier of disease severity by modifying OPN activity [78,79]. It has been recently reported that, in the *mdx* mouse, OPN exacerbates the dystrophic phenotype by skewing macrophage polarization and promoting TGF-β1 activation via matrix metalloproteinase-9 (MMP-9) [80]. This extracellular protease and its inhibitor TIMP-1 are also strongly suggested as DMD progression plasma biomarkers. In fact, high serum levels of both MMP-9 and TIMP-1 are associated with dystrophic pathology; however, only MMP-9 has been shown to increase age-dependently, thereby becoming a marker of late-stage disease in older, non-ambulant patients [81]. Importantly, although the precise mechanisms by which MMP-9 regulates dystrophic muscle regeneration are still unclear, the knock-out of MMP-9 in *mdx*^Mmp9−/−^ mice has been found to augment satellite cell proliferation and transplanted myoblast engraftment in muscles, accompanied by a significant reduction of M1 macrophages with a concomitant increase in the number of pro-myogenic M2 macrophages [82].

Several microRNAs (miRNAs) are specifically expressed in healthy skeletal muscle fibers, playing a crucial role in muscle development; DMD patients and *mdx* mice share a common altered signature of muscle-specific miRNAs [83]. Consequently, miRNAs are attracting increasing interest in recent years. In particular, bloodstream levels of specific regeneration and degeneration miRNAs have been proposed as bona fide markers for DMD diagnosis and therapeutic outcome [84]. In particular, miR-1 and miR-133, normally expressed in mature muscle fibers, are 2-fold reduced in the DMD muscle signature, whereas the levels of regeneration miRNAs, including miR-206, are doubled in satellite cells and proliferating myoblasts of dystrophic muscles. In parallel, the high serum levels of all these miRNAs in DMD patients and animal models compared to controls derive from the intensive degeneration occurring in DMD muscles. Interestingly, high levels of circulating miRNAs correspond to poor ambulant activity in patients [84].

In addition, other less canonical biomarkers of regeneration can come from functional studies at the cellular level. For instance, the expression and function of specific ion channels in myofibers may be useful indicators of the repairing process, and of activation of myogenic process and myofiber differentiation. These include various voltage-gated ion channels, such as Nav1.4, Kir, Cav1.1 [85]. One of such biomarkers we had the chance to better characterize in the frame of degeneration/regeneration events in *mdx* muscles is the macroscopic conductance to ClC-1 muscle chloride channel (gCl). ClC-1 channel is a skeletal muscle-specific channel of key importance for its role in setting sarcolemmal excitability. Its expression and function are strictly developmentally, phenotypically, and nerve regulated. The gCl is directly sensitive to inflammation, as shown by its decrease in response to inflammatory cytokines, chronic exercise, and angiotensin II (ANGII) in wt and *mdx* animals. In parallel, gCl is increased during active regeneration phases and by regeneration-promoting factors, such as IGF-1, as also shown in response to drugs with anti-inflammatory properties, such as gold standard α-methylprednisolone (PDN) [86,87,88]. The main disadvantage of functional biophysical biomarkers resides in the complex and time-consuming methodology required, which limits validation.

Finally, the evaluation of the expression of main regulators of stem cells division and polarization (e.g., partitioning-defective Par1b and Pard3) to monitor the ability of satellite cells to enter the myogenic program, maintain cell polarity, and ensure a proper asymmetric division is of the utmost importance to assess the effects of pharmacological approaches on dystrophic muscle stem cell niche balance [42,88].

Since none of these biomarkers may unambiguously identify the regenerative state in dystrophic muscles, it is essential to complementarily use these indices and to implement research to identify new ones, with the final purpose of obtaining an exhaustive view of these highly-orchestrated mechanisms, their alteration in the pathology, and the effects of drugs. In light of these observations, the following paragraphs will review the most relevant results obtained by standard therapy (i.e., glucocorticoids) and novel pharmacological approaches in DMD, with a particular focus on preclinical findings highlighting the ability of these drugs to enhance regeneration efficiency in dystrophic muscles, via the analysis of predictive biomarkers. Considering the great amount of preclinical data available on the *mdx* mouse model and the plethora of new compounds proposed and repurposed as potentially effective treatments in DMD, specific attention has been devoted to describing the impacts of drugs targeting muscle stem cell niche homeostasis in the regenerative process, particularly of those directed against pro-inflammatory and pro-fibrotic mediators, and of drugs directly aimed at directly modulating satellite cell self-renewal.

**Table 2:** Biomarkers of regeneration in DMD. List of tissue and circulating biomarkers identified for the assessment of regeneration in dystrophic animal models and also in DMD patients. The main techniques to perform their assessment at the structural and molecular level and the meaning of each biomarker in the regenerative process in relation to disease stages are also indicated.

Regenerative Biomarkers in DMD					
Biomarker	Sample Type	Detection Method	Disease Phase	Role-Meaning	References
Centronucleation and variation in fiber size	Skeletal muscle	Histology (H&E)	Early stage	Index of degeneration/regeneration cycles	*TREAT-NMD SOPs DMD_M.1.2.007, DMD_M.1.2.001;* [66,68]
Embryonic and neonatal MyHCs	Skeletal muscle	IHC, IF imaging	Differential expression depending on muscle and age	Indicator of muscle damage; correlates with functional impairment	[67]
Macrophage phenotypes (M1, M2)	Skeletal muscle	IHC, IF imaging	Early stage	Immune response during degeneration/regeneration	[68,72]
Pax7, Myf5, MyoD, Myog	Skeletal muscle	IHC, IF imaging; qRT-PCR, gene arrays; WB, ELISA, protein arrays	Differential expression depending on myogenesis stage	Myogenic regulatory factors	[68,69]
Par1b, Pard3	Skeletal muscle	IHC, IF imaging; qRT-PCR, gene arrays; WB, ELISA, protein arrays	Early stage	Regulators of stem cells asymmetric division and polarization	[42,68]
Utrophin	Skeletal muscle	IHC, IF imaging (for sarcolemmal localization); qRT-PCR; WB	Early stage	Abundant in early developing muscles and during repair	[68]
NCAM	Skeletal muscle	IHC, IF imaging	Early stage	Marks adult myogenic cells committed to differentiation	[74,75]
VEGF	Skeletal muscle	IHC, IF imaging	Early stage	Indicator of microvasculature progression	[73]
Osteopontin	Serum, Skeletal muscle	ELISA, IF imaging	Early stage	Secreted by myoblasts and macrophages after injury; correlates with disease severity	[76,77,78]
MMP-9, TIMP-1	Serum	ELISA	Late stage (age-dependent increase of MMP-9)	Remodeling of ECM; activation of latent TGF-β1; inhibition of MMP-9 increases SCs proliferation	[81,82,89]
MicroRNAs signature (miR-1, miR-133, and miR-206)	Serum, Skeletal muscle	qRT-PCR	Differential expression in plasma/muscle depending on regeneration level	Specifically expressed in muscle and released in the bloodstream as a consequence of fibers degeneration	[83,84]
Ion channel biophysics, i.e., macroscopic conductance to ClC-1 chloride channel (gCl)	Skeletal muscle	Intracellular recordings with glass microelectrodes	Early and late stages	Biophysical index directly sensitive to inflammation; increased by regeneration and anti-inflammatory drugs	[86,87]

Abbreviations: ELISA, enzyme-linked immunosorbent assay; H&E, hematoxylin and eosin; IF, immunofluorescence; IHC, immunohistochemistry; MMP-9, matrix metalloproteinase-9; Myf5, myogenic factor 5; MyHC, myosin heavy chain; MyoD, myoblast determination protein 1; NCAM, neural cell adhesion molecule; Par1b, partitioning-defective 1b; Pard3, partitioning-defective 3 homolog; Pax7, paired box protein 7; TIMP-1, tissue inhibitor of metalloproteinases 1; VEGF, vascular endothelial growth factor; WB, Western blot.

### 4.2. Glucocorticoids: Disease-Related Effects on Degeneration/Regeneration Efficiency for an Old Class

Glucocorticoids are currently the only established supportive therapy used in DMD boys at early pathology stages, although their severe side effects negatively impact on patients’ quality of life. The beneficial effects of gold standard medications (i.e., prednisone, prednisolone, deflazacort) are well documented: they control inflammation, delay pathology progression, and increase loss of ambulation up to 2 years in young DMD patients [62,64,65]. Despite this, the precise molecular mechanisms behind their ability to alleviate DMD symptoms remain largely unknown. In this context, several preclinical and clinical studies suggest that glucocorticoids may exert their primary effects by controlling muscle inflammation and fibrosis, and regeneration. We and others collected extensive evidence about the effects of PDN administration to *mdx* mice and its impact on biomarkers of regeneration (see Table 3). 

In multiple studies, we observed that treating *mdx* mice from 4–5 weeks of age with PDN (1 mg/kg; for 4 or 8 weeks), resulted in a potent anti-inflammatory action, as shown by the reduced levels of activated p65 nuclear factor-κB (NF-κB) by IHC, and in a marked reduction of reactive oxygen species (ROS), measured by dihydroethidium IF staining in dystrophic muscles. This was accompanied by a notable increase in extensor digitorum longus (EDL) myofibers gCl [86,87,90]. NF-κB modulation by PDN was also confirmed at transcript levels by qRT-PCR in other studies [91]. Importantly, PDN was also able to increase utrophin expression, measured by IF in *mdx* gastrocnemius (GC) muscle, as a direct index of improved regeneration; in parallel, α and β-dystroglycan were found increased by Western blot, as indices of improved membrane stability [71]. 

Furthermore, in a recent study by McNally and colleagues, the weekly administration of prednisone or deflazacort to *mdx* mice was associated with more consistent expression of muscle repair markers Annexins *A1* and *A6* compared to daily treatment, in parallel to a reduction of inflammatory macrophage infiltration and fibrosis, suggesting that dystrophic muscle remodeling may be also regimen-specific; therefore an appropriate dose frequency could further enhance muscle recovery and proper regeneration, possibly reducing side effects [92].

In the clinical setting, muscle biopsies from DMD patients treated with deflazacort for 3 months, gene and protein expression analyses of selected regenerative, and regulatory biomarkers showed that the drug increased the most important mediators of myogenesis and myofiber regeneration (Pax7, Myf5, MyoD, C-MET) and reduced neonatal MyHC, indicating an improved maturation process. In parallel, deflazacort strongly decreased TNF-α and macrophage-related *CD68* [93]. Accordingly, a 6-month treatment with prednisone in DMD boys was associated with an increased number of satellite cells, paralleled by a decrease in fibroblasts and dendritic cells [94]. 

Interestingly, the SNP of *SPP1* identified as a determinant of DMD disease severity has been recently associated with an alteration in response to deflazacort treatment in patients, with an increase in serum OPN levels [79,95], further supporting the existence of a cross-talk between regenerative pathways and glucocorticoids pharmacological actions in dystrophic muscles.

All these findings suggest that the effect of glucocorticoid therapy in dystrophic muscles is at least partially mediated by an improved regeneration and that this effect is “paradoxical” compared to that observed in individuals with functional dystrophin, where glucocorticoids are known to trigger muscle atrophy.

### 4.3. Pharmacological Approaches Targeting Inflammatory Mediators and Pathways

Among the new therapeutic avenues explored to pharmacologically modulate secondary events in DMD pathogenesis, several attempts have been focused on drugs potentially more effective and safer than standard glucocorticoids, targeted against pro-inflammatory molecules (see Table 4). As already stated in previous paragraphs, some of these mediators are key players of regeneration efficiency due to their direct role in muscle wasting, and then, in the modulation of functional and structural indices. For most of them, a clear impact on regeneration efficiency is lacking, and in some cases, an expected, reduction of centronucleated myofibers has been observed, as a clear consequence of necrosis.

In this general frame, a key target is the transcription factor NF-κB, a key regulator of pro-inflammatory responses in skeletal muscle. Its active form, p65 NF-κB, is highly expressed in dystrophic muscles before symptoms onset. Vamorolone (VBP15)—a Δ9,11 glucocorticoid analogue now under evaluation in Phase II clinical trials on DMD boys (NCT02760264, NCT03038399, NCT02760277) acts as a dissociative steroid, a retaining membrane, and has anti-inflammatory properties of classical steroids but loses the transactivation sub-activity associated with their side effects. First identified by Kanneboyina Nagaraju and colleagues, VBP15 strongly reduced NF-κB and TNF-α expression and percentage of inflammatory foci in 8-week-old *mdx* mice muscles, with a parallel amelioration of functional readouts [91]. A specific search of regenerative biomarkers would be certainly useful to gain more insight into the clinical efficacy of this promising therapeutic agent. Other anti-inflammatory compounds of increasing interest for DMD are edasalonexent (formerly CAT-1004, now being tested in a Phase II trial), NCT02439216 [96], and CAT-1041, two inhibitors of the IκB kinase (IKK)/NF-κB complex. In 4-week-old *mdx* mice, a 20-week treatment with each drug reduced p65 NF-κB, interleukin-6 (IL-6) and OPN protein levels, without modifying utrophin expression.

Histopathology was ameliorated, with a reduction of the total area of damage and of inflammatory macrophage infiltrates, in parallel to in vivo and ex vivo functional indices [97]. In *mdx* mice, IKK conditional deletion clarified that NF-κB functions in activated macrophages to promote inflammation and muscle necrosis, reducing regeneration via inhibition of muscle progenitor cells [98]. In 5-week-old *mdx* mice muscles, a 4-week treatment with the IKK inhibitor NEMO-binding-domain (NBD) peptide, induced strong decreases in macrophage infiltration and p65 NF-κB, measured by electrophoretic mobility shift assay (EMSA). Interestingly, increments in embryonic MyHC positive myofibers and CNF proportion, measured by IF and H&E respectively, were here taken as positive indices of increased regenerative potential, since they were accompanied by a notable inhibition of damaging pathways [99,100].

Approved drugs targeting TNF-α have been evaluated for possibly repurposing DMD, with interesting findings concerning regeneration. A 4-week treatment with etanercept (Enbrel^®^), a chimaera compound bearing the TNF-α soluble receptor, improved EDL myofibers gCl in adult *mdx* mice, while the histological profile was only modestly ameliorated. Histopathology was also analyzed in GC muscles from *mdx* mice treated from two weeks of age, when the first spontaneous degeneration cycle occurred, showing a significant reduction in the proportion of degenerating fibers; however, no clear index of regeneration was observed [101].

Several studies have been performed to assess the role of non-steroidal anti-inflammatory drugs (NSAIDs), inhibitors of cyclooxygenase (COX) enzymes, in dystrophic muscles, considering the canonical role of prostanoids in sustaining early and chronic inflammation. However, the effects of these drugs in DMD are controversial, likely in relation to the differential and tissue-specific roles of COX-related prostanoids. In fact, the various prostaglandins (PGs) have differential and opposite effects on regeneration and myogenesis, which complicate the outcomes of drugs inhibiting either or both COX-1 and COX-2 isoforms. In *mdx* mice, NSAIDs and COXIBs (e.g., ibuprofen, flurbiprofen, parecoxib) contributed to reducing macrophage infiltration to a different extent, without affecting functional indices or the percentage of regenerating myofibers. Our group could not confirm these effects for meloxicam, a COX-2 selective inhibitor, in *mdx* mice, likely in relation to the inhibition of PGE_2_, which exerts a key pro-myogenesis action [101,102]. PGD_2_, unlike other prostaglandins, inhibits myogenesis and its metabolites are increased in DMD patients; therefore, increased muscle fiber regeneration can be achieved by specific PGD_2_ inhibition. Recently, HQL-79, a potent selective inhibitor of hematopoietic PGD synthase (HPGDS), was found to suppress muscle necrosis in *mdx* mice, without interfering with cytoprotective PGE_2_ and other pro-myogenic PGs [103]. A highly-selective HPGDS inhibitor, TAS-205, was found to reduce necrosis and improve locomotor activity in *mdx* mice [104]; although no more specific results are available on its regenerative potential, a recent Phase I trial provided early evidence of a modest if any, potential therapeutic activity in DMD population (NCT02246478). 

An interesting drug target is IL-6, a myokine known to induce a harmful inflammatory milieu in human and murine dystrophic muscles, by promoting the transition from acute neutrophil infiltration to chronic mononuclear cell infiltration; however, IL-6 has also been reported to participate in muscle regeneration by promoting myoblast differentiation [105]. In 4-week-old *mdx* mice, the IL-6 pharmacological blockade via neutralizing antibody, ameliorated functional performance, modulated inflammation via NF-κB inhibition, and improved homeostatic maintenance of dystrophic muscles, as evidenced by the significant gene upregulation of *Pax7*, *MyoD1*, *Myog*, *IL-4*, and *Wnt7a*, a secreted factor which drives the “planar cell polarity pathway” to promote satellite stem cell expansion via symmetric division. In another study, IL-6 blockade increased inflammation with no functional improvement, suggesting that attention should be paid to its dual role in dystrophic muscles, concerning any possible drug intervention [106,107,108].

Additionally, compounds with anti-inflammatory properties related to multiple intracellular actions may exert clear effects on regeneration in DMD. Among these, flavocoxid, a mixed flavonoid with antioxidant and NF-κB inhibiting properties, was described to exert an early, remarkable morphological benefit evidenced by H&E staining in 5-week-old *mdx* mice muscles, by reducing necrosis and mononuclear cell infiltrate, with an increased regenerating area defined as an increase in the number of Myog-positive nuclei by IHC, while CNF were comparable to vehicle [109].

Another wide-acting drug is pentoxifylline (PTX), a non-selective phosphodiesterase inhibitor exerting anti-inflammatory, anti-cytokine, and anti-fibrotic actions linked to a specific ability to inhibit abnormal calcium entry in dystrophic myofibers [74]. We found that a 4-week treatment with PTX restored calcium homeostasis and reduced markers of oxidative stress in *mdx* mice. Histopathology and fibrosis were improved in a muscle-specific manner. Interestingly, although no significant variation in CNF percentage was observed, PTX significantly increased the NCAM-positive area in diaphragm (DIA) and GC. Then, a wide action of PTX can be envisaged: A reduction of muscle necrosis by controlling inflammation-related oxidative stress and calcium homeostasis, while stimulating regeneration via reducing pro-fibrotic signaling and activating satellite cells. In vitro experiments with PTX in C_2_C_12_ cell cultures further supported the potential ability of PTX to directly activate satellite cells and promote their growth, likely resulting from cAMP increase in satellite cells [74]. Whitehead et al. provided evidence that the anti-oxidant compound *N*-acetylcysteine (NAC) ameliorates skeletal muscle pathophysiology in GC muscles from 8-week-old *mdx* mice, by reducing ROS production, NF-κB activation, and CNF, and importantly, increasing utrophin and β-dystroglycan levels at the sarcolemma [70].

### 4.4. Pharmacological Approaches Targeting Pro-Fibrotic Mediators

TGF-β1 is a multifunctional cytokine playing a role as a master regulator of both ECM remodeling and myogenesis. In healthy muscles, a timely activation of TGF-β1 and satellite cells is thought to be critical for muscle recovery and development. In DMD patients and *mdx* mice, high levels of TGF-β1 correlate with disease severity and induce excessive collagen deposition, contributing to fibrosis progression (see Table 4). In parallel, the TGF-β1-SMAD (small mother against decapentaplegic) 2/3 pathway can also inhibit the activation of myogenic regulatory factors, thereby inhibiting proliferation and differentiation of satellite cells [13,110]. Therefore, agents able to prevent fibrosis by reducing TGF-β1 pathway, either directly or via modulation of upstream activating signals or epigenetic mechanisms, may be potentially beneficial to improving regeneration in DMD.

A main anti-fibrotic action has been attempted with halofunginone (HT-100), an anti-coccidial drug that inhibits TGF-β1 downstream signaling. This drug was described to promote satellite cell activation and survival in vitro in cultured myofibers from 6-week-old *mdx* mice, as shown by increased MyoD expression, with parallel reduction of pro-apoptotic Bax and Bcl2 [111].

As previously discussed, MMP-9 is aberrantly regulated in both DMD patients and *mdx* mice and likely involved in the cross-talk between inflammation (NF-κB activation augments MMP-9 expression) and fibrosis (MMP-9 cleaves latent TGF-β1). Early 5-week treatment with the MMPs inhibitor batimastat (BB-94) was able to significantly reduce the mRNA expression of a variety of MMPs, including *MMP-9*, *NF-kB*, *TNF-α*, and *TGF-β1* in *mdx* mice; at the histological level, batimastat-treated GC muscles showed significantly reduced fibrosis; and accumulation of macrophages, CNF, and embryonic MyHC-stained myofibers, paralleled by an increase in utrophin protein expression, measured by Western blot [89,112]. TGF-β1-mediated fibrosis and prevention of proper muscle tissue regeneration is also sustained by the increased expression of connective tissue growth factor (CTGF/CCN2) in DMD patients and *mdx* mice, where CTGF is mainly detected in regenerating fibers and in the interstitium between damaged fibers [110]. FG-3019 is an anti-CTGF antibody, found to control muscle damage and improve function in *mdx* mice after a 2-month treatment. However, the ability of FG-3019 to reduce necrosis (less uptake of Evans Blue dye) and fibrosis, implied also a concomitant reduction of regenerating activity, as shown by decreased levels of embryonic MyHC and Myog [113]. FG-3019 is now being tested in Phase II clinical trials on DMD boys (NCT02606136); again, the search of biomarkers of regeneration in patients would be useful.

The pharmacological inhibition of TGF-β superfamily member myostatin is considered as another attractive therapeutic option for DMD patients, for both increasing muscle mass and helping regeneration via reduction of the non-permissive pro-fibrotic environment [114]. Very recently, Wagner et al. demonstrated that the delivery of a myostatin inhibitor (RK35) in tibialis anterior (TA) muscle of dystrophic *mdx*-5^Cv^ mice, mediated by a biological hydrogel scaffold, was able to modulate muscle’s immune microenvironment, by promoting a pro-regenerative macrophage polarization, facilitating the M1 to M2 transition, and facilitating the consequent production of anti-inflammatory cytokine IL-10 [115].

In this general frame, also the epigenetic modulation of myostatin/follistatin axis via histone deacetylase inhibitors (HDACi) deserves attention. In particular, the HDACi givinostat was found to induce a functional improvement in vivo in *mdx* mice, and importantly, a reduction of neutrophil granulocytes evaluated by IF for myeloperoxidase in TA muscle used to quantitate the magnitude of inflammation associated with muscle degeneration [116]. In the clinical setting, a 12-month treatment with givinostat in a Phase II study on DMD boys, significantly decreased total fibrosis, necrosis, and adipose tissue replacement, in parallel to increasing myofibers size, although no direct regenerative biomarker was assessed. Now, a safety and efficacy Phase III multicentre study is ongoing in ambulant patients (NCT02851797; [117]). By the way, the ability of HDACi to enhance regeneration also via nitric oxide (NO) pathways [118] is a main working hypothesis that would deserve to be specifically verified at both preclinical and clinical levels.

Other important approaches to control dystrophic muscle fibrosis are those acting through a multifaceted mechanism or on prime signals in fibrotic pathways, such as angiotensin II-related ones [87]. In *mdx* mice, a 6-month treatment with the antihypertensive losartan, an angiotensin-II type 1 receptor blocker, was found to decrease angiotensin II-mediated TGF-β1 signaling, with marked in vivo functional improvement. At the histological level, it attenuated disease progression and improved regeneration, measured as an increase in neonatal MyHC [119]. Interestingly, we reported that early treatment in *mdx* mice with the ACE inhibitor enalapril exerted mainly an anti-oxidant and anti-inflammatory action (via NF-κB inhibition). CNF percentage was reduced in GC muscle of treated mice, while the increase in gCl of EDL muscle could be related to a reduction of the direct action of ANGII on ClC-1 channel, more than to an enhanced regeneration [86]. This underlines how the disease phase is relevant in determining a different drug response and to observe an effect on regeneration efficiency, which can be more likely to be appreciated after a long-lasting control of the niche environment.

This simple hypothesis is not fully supported by data with other drugs which may exert an anti-fibrotic action in dystrophic muscles. Metformin (MET), a well-known anti-diabetic drug, has been repurposed in combination with NO-donors in clinical trials on DMD patients (NCT01995032). Recent reports described the ability of MET to directly inhibit TGF-β1-SMAD 2/3 mediated fibrosis [120]. Accordingly, we disclosed that a 20-week treatment with MET in *mdx* mice exerted a potent, metabolism-independent anti-fibrotic action evidenced by a significant functional and structural improvement, accompanied by decreased TGF-β1 levels in GC muscle. However, no significant changes were observed on histological biomarkers of regeneration, i.e., CNF proportion. Then, it is feasible that the molecular mechanism underlying the anti-fibrotic action (still under clarification for MET) or other parallel mechanisms can define the outcome on regeneration efficiency [121]. Interestingly, Pavlidou et al. recently reported that, in C57BL/6 mice, MET delayed satellite cell activation and maintained quiescence (as shown by reduced Pax7 protein expression) [44].

In our laboratory, the effects on fibrosis and regeneration biomarkers in *mdx* mice were also investigated after a treatment of 4 or 12 weeks GLPG0492, a non-steroidal selective androgen receptor modulator (SARM), proposed as a potential anabolic therapy for DMD patients. GLPG0492 reduced fibrosis and TGF-β1 levels in DIA muscle; however, neither histological signs of regeneration nor an increase in the expression of regeneration-related genes (*Myog*, *follistatin*, or *IGF-1*) was found [122], in spite of the fact that androgen receptor modulation is supposed to enhance myogenesis [123]. In parallel, the anticancer drug tamoxifen, a selective oestrogen receptor modulator (SERM), was shown to act as a ROS scavenger and inhibitor of fibroblast proliferation. Dorchies et al. tested the effects of long-term treatment with tamoxifen in the *mdx*-5^Cv^ strain, which was found to induce a slower dystrophic phenotype, by reducing DIA muscle fibrosis and increasing CNF proportion [124]. Tamoxifen has been granted the designation of orphan drug by European Medicines Agency in 2017, and is currently under evaluation in a Phase III multicentre trial in DMD patients (NCT03354039). The apparent controversial results can be due to the different pathways modulated by the drugs that need in turn to be interpreted in the frame of the pathology-related events. Interestingly, estrogen receptor EERα in skeletal muscle is known to be an auxiliary co-activator of PGC-1α in enhancing endogenous anti-oxidant response and mitochondrial oxidative metabolism [125,126,127]. A role of the latter in satellite cells’ activation and stem cell niche has been proposed [127].

### 4.5. Pharmacological Approaches to Enhance Satellite Cell Myogenic Capacity

Besides cell-based therapies and gene replacement strategies aimed at satellite cell reprogramming in DMD, new pharmacological interventions have been recently explored to target muscle stem cell microenvironment and stimulating intrinsic repair (see Table 4). Asymmetric cell division plays a pivotal role in the maintenance of the satellite cell pool. The granulocyte colony-stimulating factor receptor (G-CSFR) is asymmetrically segregated during muscle stem cell division and the G-CSF/G-CSFR axis supports long-term muscle regeneration in mice. Filgrastim, a G-CSF analogue currently being tested for efficacy and safety in a Phase I study on DMD patients (NCT02814110), increased myocytes and improved regeneration in *mdx* mice [128]. 

A treatment with the secreted factor Wnt7a, which drives the symmetric expansion of satellite cells [129,130], resulted in increased specific muscle force and reduced contractile damage in *mdx* mice. In parallel, it induced hypertrophy and a shift toward slow-twitch in human primary myotubes [19].

β1-integrin is another essential niche molecule that maintains satellite cell homeostasis, sustaining the expansion and self-renewal of the stem cell pool during regeneration. The exogenous administration of β1-integrin enhanced regeneration in vitro and also muscle function in vivo in *mdx* mice [131].

Interesting preclinical results were also obtained via pharmacological inhibition of p38MAPK, which is aberrantly regulated in regenerating dystrophic muscles, although the exact mechanism and the link with regeneration and myogenesis remains to be better determined. Treatment with the p38MAPK-inhibitor SB731445 in the *Sgcd^−/−^* mouse model was able to ameliorate the dystrophic phenotype and to improve the self-renewal of satellite cells [132].

Finally, unacylated ghrelin (UnAG) is a circulating hormone that protects muscle from atrophy, promotes myoblast differentiation, and enhances ischemia-induced muscle regeneration. UnAG was found to reduce muscle degeneration, improve muscle function, and increase dystrophin-null SC self-renewal in *mdx* mice, maintaining the satellite cell pool [133]. These first preclinical observations support the use of drugs aiming to directly restore polarity and proper mitotic division of satellite cells, as part of DMD therapy in the future, although a larger body of evidence regarding the mechanisms underlying their effects in dystrophic muscles will be needed to improve data translatability.

**Table 4:** Pharmacological approaches targeting niche homeostasis in DMD. Synthetic overview of drugs, new or repurposed, targeting the muscle stem cell niche microenvironment in dystrophic muscles by acting on inflammation, fibrosis, or self-renewal, and of their effects on regenerative biomarkers in *mdx* mice muscles. For drugs translated into clinical settings, the stage of development in DMD patients is indicated, and for repurposed drugs, the approval for other pathological conditions. * ClinicalTrials.Gov identifiers.

Some Novel Pharmacological Strategies Potentially Targeting the Niche Microenvironment in DMD				
Drug	Molecular Target	Direct/Indirect Effects on Regeneration (*mdx* Mouse Model)	Clinical Status	References
**Inhibition of inflammation**				
vamorolone (VBP15)	NF-κB	Reduced NF-κB and TNF-α expression (qRT-PCR, IF) Reduction of inflammatory foci (H&E)	Phase II NCT02760264, NCT03038399, NCT02760277 *	[91]
CAT-1004 (edasalonexent) CAT-1041	IκB kinase/NF-κB complex	Reduced activated p65 NF-κB, IL-6 and osteopontin protein expression (WB)	Phase II (edasalonexent) NCT02439216	[96,97]
NEMO-binding-domain peptide	IκB kinase	Reduced activated p65 NF-κB (EMSA) Reduced macrophage infiltrates (H&E)	-	[99,100]
etanercept (Enbrel^®^)	TNF-α	Increased EDL myofibers gCl (electrophysiology) No direct regeneration index observed	FDA-approved for rheumatoid arthritis and psoriasis, no trials for DMD	[101]
NSAIDs and COXIBs	COX1 and/or COX2	Reduced macrophage infiltrates (H&E) No confirmed effects on regeneration Meloxicam: possible interference with cytoprotective prostaglandin PGE_2_	Anti-inflammatory agents	[101,102]
HQL-79, TAS-205	hematopoietic prostaglandin D synthase	Suppressed muscle necrosis (H&E) No interference with myogenic PGs No specific results available on their regenerative potential	Phase I (TAS-205) NCT02246478	[103,104]
IL-6 neutralizing antibody	IL-6	Improved the homeostatic maintenance (upregulation of *Pax7*, *MyoD1*, *Myog*, *IL-4*, and *Wnt7a* gene expression) Increased inflammation with no functional improvement also reported	-	[106,108]
IL-1Ra anakinra (Kineret^®^)	IL-1β pathway	No significant modification of disease-related regenerative parameters	FDA-approved for arthritis	[134]
flavocoxid	COX1, COX2, 5-lipoxygenase	Reduced necrosis and macrophage infiltrates; no variation in CNF percentage (H&E) Increased number of Myog-positive nuclei (IHC)	-	[109]
pentoxifylline	phosphodiesterase enzymes	Improved histopathology with no variation in CNF percentage (H&E) Increased NCAM-positive area (IHC) Increased cAMP in satellite cells in vitro	Antithrombotic agent	[74]
N-acetylcysteine	wide anti-oxidant action	Reduced NF-κB activation and ROS Reduced CNF percentage (H&E) Increased utrophin and β-dystroglycan levels at sarcolemma	Mainstay therapy for acetaminophen toxicity	[70]
**Inhibition of Fibrosis**				
halofunginone (HT-100)	TGF-β1 signalling	Promoted satellite cell activation (increased MyoD protein expression) and survival (reduced Bax, Bcl2 protein expression) in vitro (IF, WB)	Anti-coccidial agent	[111]
batimastat (BB-94)	MMP-9	Reduced mRNA expression of *MMP-9*, *NF-kB*, *TNF-α* and *TGF-β1* (qRT-PCR) Reduced MMP-9 enzymatic activity Reduced fibrosis, macrophage infiltrates and CNF (H&E, Sirius Red) Reduced embryonic MyHC and increased utrophin expression (WB)	Anticancer agent	[89,112]
FG-3019 antibody	CTGF	Reduced muscle necrosis (Evans Blue) Decreased regeneration (lower levels of embryonic MyHC and Myog)	Phase II NCT02606136	[113]
RK35	myostatin	Biological scaffold–mediated delivery Promoted M1 to M2 macrophage transition and increased IL-10 release (IHC, IF, qRT-PCR, in vivo/in vitro assays)	-	[115]
givinostat	histone deacetylase (HDAC)	Reduction of neutrophil granulocytes (IF for myeloperoxidase) In DMD boys: successfully completed Phase II study; no direct biomarker of regeneration assessed	Phase III NCT02851797	[116,117,118]
losartan	ANG II type 1 receptor blocker	Decreased ANG II-mediated TGF-β1 signalling pathway Increased neonatal MyHC (H&E, IF)	Antihypertensive agent	[119]
enalapril	angiotensin-converting enzyme	Early treatment reduced CNF (H&E) Increased EDL myofibers gCl	Antihypertensive agent	[86]
metformin	AMPK	Decreased muscular TGF-β1 (ELISA) No changes in structural regenerative biomarkers (e.g., CNF proportion) Maintained quiescence and reduced Pax 7 in healthy mice (IF, WB)	Phase III NCT01995032	[44,121]
GLPG0492	androgen receptor	Reduced muscular TGF-β1(ELISA) No increase of *Myog*, *follistatin* or *IGF-1* (qRT-PCR)	-	[122]
tamoxifen	oestrogen receptor	Reduced muscle fibrosis and increased CNF proportion (H&E)	EMA Orphan Drug Designation (2017) Phase III NCT03354039	[124]
**Promotion of Self-renewal**				
filgrastim (G-CSF analogue)	G-CSFR	Increased satellite cells and Pax 7 (IF)	Phase I NCT02814110	[128]
Wnt7a	activation of “planar cell polarity pathway”	Hypertrophy and slow-twitch fiber shift (in human myoblasts cultures)	-	[129]
β1-integrin	MAPK Erk, AKT	Enhanced regeneration in vitro Maintained the responsiveness of the niche to Fgf2	-	[131]
SB731445	p38MAPK	Treatment in the *Sgcd^−/−^* dystrophic mouse model improved satellite cells self-renewal	-	[132]
unacylated ghrelin	GHS-R; pleiotropic, tissue-specific hormonal activity	Reduced muscle degeneration Preserved the satellite cell pool at later stage of pathology	-	[133]

Abbreviations: ANG II, angiotensin II; AMPK, AMP-dependent protein kinase; Bax, Bcl-2-associated X protein; Bcl2, B-cell lymphoma 2; CNF, centronucleated fibers; COX1, COX2, cyclooxygenase 1 and 2; COXIBs, cyclooxygenase 2 inhibitors; CTGF, connective tissue growth factor; EMSA, electrophoretic mobility shift assay; Fgf2, fibroblast growth factor 2; G-CSFR, granulocyte colony stimulating factor; GHS-R, growth hormone secretagogue receptor; IL-1Ra, interleukin 1 receptor antagonist; NSAIDs, nonsteroidal anti-inflammatory drugs; PG, prostaglandin; ROS, reactive oxygen species; Wnt7a, wingless-type MMTV integration site family, member 7A.

## 5. Discussion

Satellite cells are muscle-committed stem cells resident in skeletal muscle, importantly contributing to muscle growth and differentiation, and allowing an efficient repair process after damage. Multiple intrinsic and extrinsic factors are involved in the orchestration of this complex process, thereby fascinating scientists for potential applications in the field of regenerative medicine, and for developing drugs able to counter progressive muscle wasting disorders, by enhancing an efficient repairing process in inherited or acquired conditions. DMD is a prototype of the efforts in this field, due to the intense research aimed at identifying potential therapies. DMD in fact has no cure; the progressive muscle degeneration is directly related to the events following the primary defect, which are related in a complex cross-talk, to the inefficient regeneration process. Accordingly, satellite cells are believed to be pivotal in determining disease outcome, since the exhaustion of the satellite cell pool causes the absence of a turning point of this muscle disorder. Intrinsic defects of satellite cells, due to the absence of dystrophin, have been described, and the role of the niche in the exhaustion of the satellite cell pool is still debated. As a matter of fact, the niche environment seems to be paramount in the ability of satellite cells to repair the continuous cycles of degeneration/regeneration happening in DMD [15,17,20]. Satellite cells’ niche is disrupted over time by the inflammation and fibrosis occurring as the disease progress. Therefore, while the effort to treat the primary defect is still the main target of the scientific community, drugs able to act on muscle environment deserve to be taken into consideration. This approach concerns both novel drugs and repurposed ones, the latter having the additional advantage of a more rapid translational potential from bench to clinic. Drugs with the best clinical potential would ideally target main pathogenic events, reducing damage in parallel, making repair efficient. Knowing all that, we took advantage of the large amount of preclinical data obtained in our and other laboratories with the main aim of summarizing the available evidence of a potential drug action on regenerative efficiency via a direct or indirect action on stem cell niche and satellite cells.

As reviewed here, there are many promising compounds able to improve muscle regeneration, and even glucocorticoids have a positive outcome in improving myofiber regeneration and enhancing the maturation process, mostly in relation to the regimen approach, which may in part account for their clinical efficacy. Importantly, the complex and not fully clear action of glucocorticoids in dystrophic muscle, and mostly in the satellite cell niche, deserves to be better investigated, as these drugs are able to counter degeneration while sustaining the regeneration process.

In particular, drugs acting on different ILs and other inflammatory cytokines are promising [90,100,104] and should be investigated in more depth. These drugs can take advantage of studies performed in DMD patients, and possibly gain better insight by looking at biomarkers of regeneration. Importantly, great progress has been made to identify reliable non-invasive biomarkers of both pathology progression and regeneration and drug efficacy. These will greatly help the translational assessment of therapy efficacy in the stem cell niche [42,66,67,68,69,70,71,72,73,74,75,76,77,78,79,80,81,82,83]. In a general situation, even if it is not clear yet whether is the exhaustion of the satellite cell pool or niche disruption drives the pathological progression, we know that drugs acting either on the amelioration of the niche environment or satellite cells’ asymmetric division could be good candidates to slow down DMD.

At the same time, what we have learned from a complex disease such as DMD is that the outcome of drug action on regeneration efficiency is not always straightforward in spite of robust hypotheses and a clear mechanism of action. Interestingly, among novel potential therapies, our analysis of literature data enlightened that drugs purely directed against inflammatory molecules (e.g., TNF-α, NF-κB) are mostly able to reduce muscle damage but not improve regeneration, whilst several therapeutic interventions inhibiting or modulating molecules with a pleiotropic action seem to positively impact regenerative biomarkers, in parallel with controlling damage. These molecules, i.e., myokine IL-6, pro-fibrotic TGF-β1, c-AMP dependent pathways and estrogen receptors, and all self-renewal mediators (e.g., G-CSF, Wnt7a, β1-integrin, p38MAPK, ghrelin derivatives), represent potential therapeutic targets of new/repurposed drugs for DMD to specifically support regeneration efficiency, via a direct action on satellite cells or by improving niche environment. In parallel, the effects on regeneration via the pharmacological modulation of other promising targets, e.g., HDACs and ANGII pathways, need to be further explored. In particular, this topic will grant further insight into the specific roles in the regeneration process of different HDAC isoenzymes, and roles in alternative pathways of the RAS system, such as those mediated by ANG 1-7 via MAS receptor [86,87,118].

Then, we still need to understand key aspects of this multi-actor process that in turn have to be approached at different levels. In fact, there are major key players in disease progression and homeostasis maintenance, and it is reductive to assess that the disease progression is caused by the exhaustion of the satellite cells’ niche. Then, combined strategies may work in synergy and such synergy may also occur with innovative molecular or cell therapies able to restore dystrophin expression. In fact, drugs able to address regeneration efficiency, although they will not cure, create ideal circumstances to sustain therapies aimed at correcting primary DMD defects, by creating a healthier environment. This has not been done yet, and the possible outcomes are not predictable, although they would ideally be of high clinical relevance.

In addition, the possibility to enhance our understanding of drug targetable events in myogenesis may also help to improve the in vitro approach of tissue engineering for building up both experimental platforms for drug discovery and simulation of reparative medicine.

## Figures and Tables

**Table 1:** Cytokines involved in satellite cell regeneration. List of cytokines involved in the inflammatory pathway for muscle regeneration and maintenance. The listed cytokines take part either in proliferation or differentiation of the satellite cell population aiming to repair muscle tissue after injury. Their role in relation to the phase of regeneration is indicated.

Cytokines	Effects on Satellite Cells (Early Phase)	Effects on Myoblasts (Later Phases)	References
IL-1β	Pro-inflammatory; increases SCs proliferation and coordinates interactions between SCs and microenvironment	Reduces myogenic differentiation	[23,49]
IL-4	Improves myoblast differentiation in vitro and increases Myog expression	Plays a role in SCs fusion and growth	[50]
IL-6	Pro-inflammatory; induces SCs proliferation	Stimulates hypertrophy and promotes myoblast differentiation	[51]
IL-7	None reported	Possible involvement in inhibiting differentiation (limited data available)	[52]
IL-10	Anti-inflammatory, counteracts IL-6; no effects on proliferation	Stimulates differentiation	[53]
IL-13	Pro-inflammatory; increases SCs proliferation	Fusion-promoting activity	[48,54]
IFN-γ	Pro-inflammatory; increases SCs proliferation	Impairs differentiation via inhibition of Myog expression	[48]
TGF-β1	Pro-fibrotic; maintains and induces SCs quiescence	Inhibits differentiation	[55]
TNF-α	Pro-inflammatory; increases SCs proliferation, activates SCs to enter the cell cycle via p38 MAPK activation	Inhibits differentiation and fusion	[23]

Abbreviations: SCs, satellite cells; IL, interleukin; IFN-γ, interferon γ; Myog, myogenin; TGF-β1, transforming growth factor β1; TNF-α, tumor necrosis factor α.

**Table 3:** Glucocorticoid therapy and degeneration-regeneration efficiency in DMD. Preclinical and clinical observations collected about the impact of glucocorticoid supportive treatments on biomarkers of regeneration in DMD. The observed direct and indirect effects on the regenerative process are briefly listed, and the techniques used for the detection.

Standards of Care for DMD and Regeneration		
Glucocorticoid Drugs	Direct/Indirect Effects on Regenerative Biomarkers	References
**α-methyl-prednisolone (PDN)** 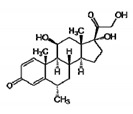	4—8 weeks of treatment in ***mdx* mice** (from 4 weeks of age) Reduced NF-κB expression and activation (qRT-PCR, IHC) Increased utrophin expression (IF) Increased EDL myofibers gCl (electrophysiology) Increased α- and β-dystroglycan (WB) Reduced macrophage infiltration (H&E)	[71,86,90]
**prednisone** 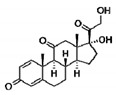	Weekly treatment in 6-month-old ***mdx* mice** (for 4 weeks) increased expression of Annexins *A1, A6* (gene arrays) 6-month treatment in **DMD patients** increased muscle satellite cells, reducing fibroblasts and dendritic cells (H&E)	[92,94]
**deflazacort** 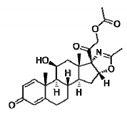	Weekly treatment in 6-month-old ***mdx* mice** (for 4 weeks) increased expression of Annexins *A1, A6* (gene arrays) 3-month treatment in **DMD patients** increased the gene and protein expression of Pax7, Myf5, MyoD, C-MET and reduced neonatal MyHC, TNF-α and macrophage-related CD68 (qRT-PCR, IHC)	[92,93]

Abbreviations: c-MET, tyrosine-protein kinase Met; NF-κB, nuclear factor kappa-light-chain-enhancer of activated B cells.
